# Biventricular Pacing in Congestive Heart Failure

**Published:** 2002-04-01

**Authors:** Balbir Singh

Congestive heart failure (CHF) is a common, debilitating and usually lethal condition responsible for enormous burden on health care. The recent trials have shown improvement in morbidity and mortality with ACE inhibitors and beta blockers [1,3]. However, despite this the overall prognosis remains dismal. The other alternative of cardiac transplantation has several limitations including the donor organ scarcity. In view of this gloomy prognosis, several alternative strategies are being explored, one of which has been pacing for heart failure. In this form of therapy pacing is used in absence of the conventional bradyarrhthymic  indications with an attempt to lead to optimization of AV delay and co-ordination of ventricular contraction.

In 1990, Hochleitner et al [4] reported clinical improvement in patients with severe heart failure awaiting cardiac transplantation with implantation of a physiologic dual-chamber pacemaker (pacing at right atrium and right ventricle) with a programmed short atrioventricular (AV) delay. Brecker et al [5] in 1992 reported similar observations. However, in a randomized cross over design,with larger number of patients there was no significant improvement in the NYHA class or ejection fraction [6]. Sack et al [7] and Guide et al reported similar negative results. Consequently, it is difficult to advocate dual chamber pacing for heart failure management.

The reasons for the discrepancies in the results of these studies is possibly due to the detrimental effect of pacing induced broadening of the QRS complex duration in severe ventricular disease resulting from Right Ventricular (RV) apical pacing offsetting the beneficial effect of increased ventricular filling time. As a result the focus has now shifted to Left Ventriuclar (LV) or biventricular (BiV) as opposed to RV pacing supplemented with the lessons learnt from the optimization of the AV delay.

## Electromechanical Cardiac Synchrony

The association of asynchronous ventricular contraction with ventricular dysfunction has been recognized for many years. In recent years the presence of left bundle branch block (LBBB) has been shown to correlate with decreased LV function, reduced peak dp/dt. LBBB results in asynchronous ventricular contraction with the LV lateral wall contracting much later that the inverventricular septum in addition there is an RV-LV asynchrony with RV contracting earlier than LV.

The presence of conduction disturbances is seen in 20-30% of the patients with congestive heart failure and contributes to the worsening of symptoms due to improper co-ordination of LV contraction.

## Cardiac Resyndronisation Therapy (CRT)

CRT aims at 3 different levels (a) AV level (b) intraventricular level (c) interventricular level. At present this is achieved by pacing or sensing the right atrium, pacing the right ventricle (near the interventricular septum) and pacing the left ventricle (using the coronary venous branches), also called biventricular pacing.

## Left Ventricular Lead Design

The present LV leads have lower profile with preformed curves. Most of the leads followed the same conventional central-stylet technology, with curves being fashioned to negotiate the variabilities in cardiac vein anatomy.  Recently over-the-wire lead deployment systems have been developed (Easytrak - Guidant Corporation, St. Paul, MN) and have the procedure like an angioplasty. Overall, the success rate for implantation of left-sided leads ranges from 75 to 93%.

## Implantation Technique

The implantation of biventricular pacing is more technically challenging than a dual chamber pacing for the reason of placing the LV pacing lead appropriately. Prior to the introduction of the endocardial LV pacing leads, surgical implantation of these leads epicardially was the norm.

It is now possible to pace by entering the cardiac veins which are approached through the coronary sinus and obtain a reasonable threshold in one of the cardiac veins. The presence number, location, size and tortousity of posterior and lateral branches is usually variable. The coronary veins are thus studied by contrast injections with a balloon inflated catheter within the coronary sinus and subsequently the lead can be placed precisely. The posterolateral veins yield the best haemodynamic outcome and are the ones targeted for the placement of LV leads.

The findings from the PATH - CHF trial [9] suggest that increases in pulse pressures and DP/DT max were maximum at the mid lateral epicardial pacing sites compared with other regions of the left ventricle, consequently posterolateral sites are currently targeted for left ventricular pacing. Kass et al [10] in 1999 demonstrated that LV single site pacing was equal or superior to biventricular pacing. Further studies would be needed to demonstrate whether LV pacing is equivalent. It is possible that LV pacing may not maintain LV/RV syndrony due to earlier pacing of the LV site.

## Identifying patients likely to respond

The primary variable has been the QRS duration - an electrial marker for spatially dispersed mechanical activation [8]. A QRS duration of more than 150ms with class 3 or 4 congestive heart failure and low left ventricular ejection fraction is an accepted indication for left ventricular pacing. In presence of QRS duration of 120-150msec, certain echo - doppler variables must be noted (such as Q to aortic flow velocity or interventricular dysynchrony - Q to aortic flow - Q to pulm flow) to obtain the maximum benefit.

## Acute Clinical Studies

Several studies have studied the effects of BiV pacing on the acute haemodynamics. It has been seen that BiV pacing improves cardiac output and enhances ventricular systolic function as assessed by maximal rate of pressure rise and pressure volume loops. Furthermore this improvement in LV systolic function occurs while concomitantly reducing myocardial oxygen consumption.

## Chronic Clinical Studies

Three placebo control studies have been completed - the PATH - CHF trial [9], the MUSTIC trial [11] and the MIRACLE trial [12]. In the PATH-CHF study, patients were first assigned to four weeks of active pacing (LV or BiV), then four weeks of no pacing, then a second four-week active pacing period-continued for the ensuing year. This was a single-blind study and required surgically implanted leads and two stimulators. Importantly, exercise performance (e.g., maximal oxygen consumption) rose significantly only during the two periods of active pacing. This finding in the third month (after a month of no-pacing) was somewhat more difficult to ascribe to a placebo effect.

The recently published MUSTIC study [11] used a cross-over design, with patients randomized to three months' stimulation on or off and the mode then switched for the second three-month period. In sinus rhythm patients, exercise capacity improved olnly during active treatment (+23% in 6-min walking distance, p < 0.001), improved symptoms (32% in quality-of-life questionnaire, p < 0.001) and increased maximal oxygen consumption (+8%, p < 0.03). Interestingly, this study did not observe a placebo effect. A separate component of this study evaluated patients with chronic atrial fibrillation, each patient under-going AV nodal ablation prior to receiving a BiV stimulation system. Intention-to-treat analysis failed to reveal significant differences between pacing on and off data, although limitations due to study design and loss of effective pacing in several subjects contributed to this. In the subset of subjects in which pacing was effectively delivered, the results suggested improvement, but this needs more definitive testing.

The recently completed MIRACLE trial [12] is the largest study to date. Preliminary data have been reported and a full publication is pending. This six-month parallel-design trial randomized 228 patients to resynchronization therapy and another 225 patients to a placebo control arm. All patients were in normal sinus rhythm and were stable NYHA functional class III or class IV. The primary findings showed an improvement in the 6-min walk test, quality-of-life score, and NYHA functional class (a combined end point was also examined). Secondary end points were also assessed in a subset of patients, and the data support a diminished diastolic and systolic chamber size in the active resynchronization treatment but not in the placebo group. Mortality was <10% in both treatment arms at six months. Rehospitalization rates and number of days hospitalized were both significantly and substantially lower in the active treatment group. The investigators reported a placebo effect with respect to quality of life but not for exercise or cardiac-function parameters.

## Unresolved Issues, Future Directions

Clearly, there are major important questions about whether there is a sustained benefit on morbidity and reduced hospitalization and whether there is a favorable effect on overall and cardiac mortality. In this regard, it is important that the ongoing trails such as COMPANION, which are addressing these key questions, proceed to completion so that the role of this therapy can be properly and fully evaluated. The mortality impact of resynchronization may ultimately be tied in with ICDs, particularly if the results of ongoing multicenter trials show survival benefits from such devices in HF.

Another question relates to the prospective identification of responders. New methods examining regional wall motion hold promise for generating a dyssynchrony index that could improve on current, more indirect methods. The optimal method of therapy itself is unresolved. As noted, questions remain as to whether BiV stimulation is needed, whether multisite left-heart stimulation would enhance the efficacy, or, if an RV lead is to be placed, where the optimal location is and what the best timing delay is between RV and LV stimulation.

A large unresolved question is whether this therapy is going to be useful in patients with atrial fibrillation. Some studies have suggested utility, although larger trial data remain inconclusive. Unlike the sinus rhythm patients, in which there is some degree of freedom in the AV delay to optimally time a resynchronization effect, the AV node in atrial fibrillation patients is generally ablated, and then patients are treated using a BiV pacing mode. This involves regularization of the heart rate with rate-responsive generators, as well as activation of both lower chambers. Rate response serves to simulate normal effects of autonomic tone, but it is not a perfect replacement for physiologic control. Furthermore, in patients without an existing conduction delay, BiV pacing may not yield as good a response as that with His-Purkinje conduction. More studies are clearly needed in these patients.

Finally, the existing evidence indicating deterioration of systolic function and energetic efficiently with pacing-induced dyssynchrony suggests that standard RV apex pacing in individuals with cardiac failure may not be the ideal approach. In patients with cardiodepression but a narrow QRS complex and normal intraventricular conduction who need pacing for rate control, a BiV system may prove superior, but this clearly needs to be tested.
